# Seventh Pandemic *Vibrio cholerae* O1 Sublineages, Central African Republic

**DOI:** 10.3201/eid2701.200375

**Published:** 2021-01

**Authors:** Sebastien Breurec, Thierry Franck, Elisabeth Njamkepo, Jean-Robert Mbecko, Jean Rauzier, Hugues Sanke-Waïgana, Guyguy Kamwiziku, Renaud Piarroux, Marie-Laure Quilici, François-Xavier Weill

**Affiliations:** Institut Pasteur de la Guadeloupe, Les Abymes, France (S. Breurec); INSERM Centre d’Investigation Clinique 1424, Pointe-à-Pitre/Les Abymes, France (S. Breurec);; Université des Antilles, Pointe-à-Pitre, France (S. Breurec);; Institut Pasteur de Bangui, Bangui, Central African Republic (S. Breurec, T. Franck, J.-R. Mbecko);; Institut Pasteur, Paris, France (E. Njamkepo, J. Rauzier, M.-L. Quilici, F.-X. Weill);; Université de Kinshasa, Kinshasa, Democratic Republic of the Congo (G. Kamwiziku);; Assistance Publique-Hôpitaux de Paris, Paris (R. Piarroux)

**Keywords:** *Vibrio cholerae* O1, cholera, Central African Republic, genomics, outbreak, bacteria, enteric infections, waterborne infections, foodborne infections, food safety

## Abstract

Four cholera outbreaks were reported in the Central African Republic during 1997–2016. We show that the outbreak isolates were *Vibrio cholerae* O1 serotype Inaba from 3 seventh pandemic El Tor sublineages originating from West Africa (sublineages T7 and T9) or the African Great Lakes Region (T10).

Cholera is a life-threatening diarrheal disease caused by the bacterium *Vibrio cholerae*, which produces cholera toxin. The seventh cholera pandemic, caused by *V. cholerae* O1 biotype El Tor, began in Indonesia in 1961 and reached Africa in 1970 (*1*). Fifty years later, >100,000 cases of cholera are reported annually in sub-Saharan Africa (*2*). 

The Central African Republic (CAR) is a landlocked and resource-limited country in central Africa; it was ranked 188/189 on the United Nations Human Development Index in 2018 (http://hdr.undp.org). CAR is relatively large but has a low population density; 2019 data estimate ≈4.75 million inhabitants, or 7.75 persons/km^2^ (Macrotrends LLC, https://www.macrotrends.net). CAR largely has been spared by the cholera epidemic; only 4 outbreaks had been reported by 2020 (Table 1; Figure 1). The first 2 cholera outbreaks occurred during the same month in 1997 (*4*). In the first, 443 cases and 88 deaths were reported in southern CAR, along the Oubangui River, close to the border with the Democratic Republic of the Congo (DRC). In the second, 113 cases and 19 deaths were reported in northern CAR, close to the borders with Cameroon and Chad, after which cholera cases continued to be detected in southern CAR, along the Oubangui River. According to reports from the International Federation of Red Cross and Red Crescent Societies, 172 cholera cases and 16 deaths were reported in the region in 2011 (https://reliefweb.int/sites/reliefweb.int/files/resources/MDRCF009finrep.pdf) and 265 cases and 20 deaths were reported in 2016 (https://reliefweb.int/report/central-african-republic/central-africa-republic-cholera-epidemic-outbreak-dref-operation). 

**Table 1:** Characteristics of the *Vibrio cholerae* O1 isolates associated with outbreaks of cholera, Central African Republic*

Characteristics	Outbreak no. 1, 1997 Jun–Oct	Outbreak no. 2, 1997 Jun–Aug	Outbreak no. 3, 2011 Sept–Oct	Outbreak no. 4, 2016 Jul–Dec
No. deaths/no. cases†	88/443	19/113	16/172	20/265
No. isolates	9	6	7	8
7PET sublineage	T7	T9	T10	T10
Sequence type	69	69	515	515
*ctxB*	B3	B1	B1	B1
*wbeT*‡	A03	C19	B01	B01
AMR phenotypes (no. isolates)§	R1 (8), R2 (1)	R3 (6)	R3 (7)	R3 (8)
AMR determinants				
Plasmid	IncA/C¶	NT	NT	NT
VC_0715	WT	R169C	R169C	R169C
VC_A0637	WT	Q5Stop	Q5Stop	Q5Stop
SXT/R391 element	NT	ICE*Vch*Ind5	ICE*Vch*Ind5	ICE*Vch*Ind5
* gyrA*	WT	WT	S83I	S83I

*7PET, seventh pandemic *Vibrio cholerae* O1 El Tor; AMR, antimicrobial resistance; NT, not detected; WT, wild type. †Data from the International Federation of Red Cross and Red Crescent Societies 2011 (https://reliefweb.int/sites/reliefweb.int/files/resources/MDRCF009finrep.pdf) and 2016 (https://reliefweb.int/report/central-african-republic/central-africa-republic-cholera-epidemic-outbreak-dref-operation). ‡Nomenclature according to Weill et al. (*3*) (Table 2). §R1, resistance to polymyxin B and colistin; R2, resistance to polymyxin B, colistin, ampicillin, streptomycin, sulfonamides, vibriostatic agent, trimethoprim/sulfamethoxazole, chloramphenicol; R3, resistance to polymyxin B, colistin, streptomycin, sulfonamides, vibriostatic agent, trimethoprim/sulfamethoxazole, furazolidone, and resistance or intermediate resistance to chloramphenicol. ¶Isolate (CNRVC970079) with the R2 AMR type.

**Figure 1:**
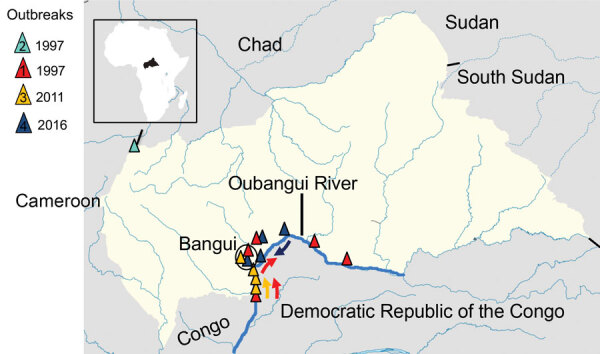
Geographic location of cholera cases during the 4 outbreaks reported in the Central African Republic, 1997–2016. Inset shows the location of Central African Republic in the continent of Africa. Numbers correspond to outbreaks during 1) June–October 1997; 2) June–August 1997; 3) September–October 2011; and 4) July–December 2016. Arrows show movement of outbreaks corresponding to colors for each outbreak

The Institut Pasteur de Bangui in CAR performed microbial analyses to confirm the causal agent of these outbreaks and identified 30 *V. cholerae* O1 serotype Inaba isolates collected during 1997–2016 (Appendix 1 Table 1). We used whole-genome sequencing to fully characterize all 30 *V. cholerae* O1 isolates in terms of virulence and antimicrobial resistance determinants. We also placed these genomes within a broader phylogenetic context to elucidate their origins and evolutionary history.

## The Study

**Figure 2:**
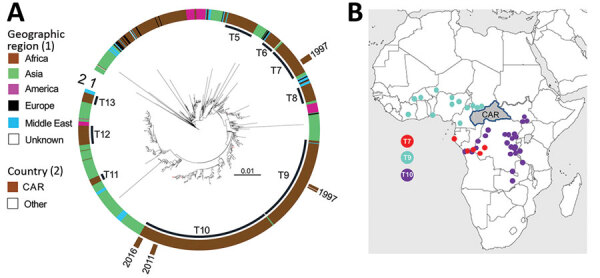
Phylogenomics of the *Vibrio cholerae* O1 El Tor isolates from CAR, 1997–2016. A) Maximum-likelihood phylogeny for 1,215 7PET genomic sequences. A6 was used as an outgroup. The last 9 sublineages introduced into Africa (T5–T13) are indicated. On inner ring, color scale denotes geographic locations of the *V. cholerae* isolates. On outer ring, brown denotes isolates from CAR. Tree branches containing isolates from CAR are shown in red. Scale bar indicates substitutions per variable site. B) Locations on the African continent in which T7, T9, and T10 *V. cholerae* O1 serotype Inaba isolates were detected before their identification in CAR. CAR, Central African Republic.

The 30 *V. cholerae* O1 isolates were received at the Institut Pasteur, Paris, France. We performed antimicrobial susceptibility testing, whole-genome sequencing, comparative genomics, and phylogenetic analyses by using methods previously described (*3*,*5*–*11*) (Appendix 2). We then contextualized these 30 *V. cholerae* O1 isolates within a global collection of 1,185 seventh pandemic *V. cholerae* El Tor (7PET) genomic sequences and constructed a maximum-likelihood phylogeny of 1,215 genomes by using 9,964 single-nucleotide variants (SNVs) evenly distributed over the nonrepetitive, nonrecombinant core genome (Figure 2, panel A). Our phylogenomic analysis (Appendix 1 Tables 2–4) showed that all CAR isolates belonged to the 7PET lineage (*12*,*13*). 

Previous genomic studies described 12 introductions of the 7PET lineage from Southern Asia into Africa during 1970–2016 (*3*,*6*). The introduced sublineages were called T1 and T3–T13. The 2 cholera outbreaks in CAR in 1997 were caused by sublineage T7, which had been introduced into West Africa during the early 1980s, and T9, which was introduced in the late 1980s, according to Weill et al. (*3*). T9 isolates were identified in neighboring countries such as Chad and Cameroon (particularly northern Cameroon) before they were detected in northwest CAR, but T7 isolates were identified in Gabon and the western part of DRC, along the Congo River in Kinshasa, before being identified in CAR along the Oubangui River, a tributary of the Congo River (Figure 2, panel B; Appendix 2 Figures 1, 2).

The 2011 and 2016 outbreaks were caused by closely related bacterial populations from the same sublineage, T10, introduced into East Africa during the 1990s and later detected in the African Great Lakes Region (AGLR) (*3*) (Figure 2, panel B; Appendix 2 Figure 3). The prevalent T10 sublineage has several clades, and the 2011 and 2016 CAR isolates are characterized by multilocus sequence type (ST) 515, a single-locus variant of ST69; ST69 is the predominant ST among 7PET isolates. In addition, these isolates have an alteration to the *wbeT* gene, a 4-nucleotide deletion called B01 (Tables 1,2), that underly the Inaba serotype. Phylogenetic data showed that the 7PET strains causing the 2011 and 2016 cholera outbreaks in CAR spread from AGLR to the western part of the DRC and CAR (Figure 2, panel B; Appendix 2 Figure 3). These genomic data are consistent with results from a recent epidemiologic study showing the spatiotemporal distribution of cholera cases during the 2011–2012 and 2015–2017 outbreaks in DRC, suggesting a spread from cholera hotspots in AGLR to major cities in the upstream section of the Congo River, followed by a downstream spread toward the densely populated CAR capital, Kinshasa, and then to the mouth of the Congo River, which opens into the Gulf of Guinea (*14*).

**Table 2:** Alterations of the *wbeT* (formerly *rfbT*) gene in *Vibrio cholerae* El Tor isolates from the Central African Republic*

Alteration no.	Alteration type	Genetic alteration	Protein consequence†	7PET sublineage
A03	Premature stop codon	G133T	G45-to-STOP	T7
B01	Premature stop codon	Deletion TGTAC (nt 24–28)	Frameshift after N7; then STOP	T10
C19	Amino acid substitution	G674A	C225Y	T9

*7PET, seventh pandemic *Vibrio cholerae* O1 El Tor. †The codon number and single-letter amino acid abbreviation are shown (STOP corresponds to a stop codon).

Except during the outbreak in northwest CAR in 1997, all cholera cases were reported along or close to the Oubangui River, suggesting that 7PET strains probably moved from area to area along the river and with the displacement of human populations. The risk factors in these remote areas are unknown, but the prevailing conditions, such as poor hygiene and sanitary conditions, overcrowding, lack of latrines, and drinking water from the Oubangui River, likely would increase the risk for transmission via the fecal–oral route, as evidenced by the high attack rates observed at several sites when the 2011 outbreak began (*15*). Nevertheless, since the declaration of the first case in 1997, the small number (<1,000) of cholera cases in CAR contrasts with the much larger numbers in central Africa (*2*). The low population density of CAR, its poor transport infrastructure, and poor trading links are probably key factors limiting disease spread (*15*). Phylogenetic analyses showed no other isolates from Africa were derived from CAR isolates in the aftermath of the 4 outbreaks, which also suggests that the transmission of cholera is impeded in this country. Of note, all 4 outbreaks were caused by serotype Inaba 7PET strains. This serotype has a nonmethylated form of lipopolysaccharide caused by an alteration to the *wbeT* gene (*3*) (Table 2). The implication of this serotype in all 4 outbreaks suggests that these 7PET sublineages circulated regionally for some time, long enough to acquire this alteration to the *wbeT* gene, before reaching CAR (Appendix 2 Figures 1–3).

All CAR isolates in this study displayed resistance to polymyxin B, consistent with the susceptibility pattern reported for the El Tor biotype until recently (*6*). All but 1 of the isolates collected along the Oubangui River in 1997 were susceptible to all other antimicrobial drugs tested; the outlying isolate contained the extended-spectrum β-lactamase *bla*_SHV-2a_ gene on an IncA/C2 plasmid (Table 1; Appendix 1 Table 3). No susceptible isolates have been collected in CAR since. All the other isolates display mutations of the *VC_0715* and *VC_A0637* genes, conferring nitrofuran resistance, and carry an SXT/R391 genomic element called ICE*Vch*Ind5, encoding resistance to streptomycin (*strAB*), sulfonamides (*sul2*), trimethoprim, the O/129 vibriostatic agent (*dfrA1*), and trimethoprim-sulfamethoxazole (*sul2* and *dfrA1*). The 2011 and 2016 CAR isolates also recently acquired *gyrA* mutations (Table 1), resulting in resistance to nalidixic acid (Appendix 1 Table 3).

## Conclusions

Strains from 3 7PET sublineages caused 4 cholera outbreaks identified in CAR during 1997–2016. The southern and southeastern parts of CAR are higher risk areas for cholera outbreaks, particularly when cases are reported in the western part of DRC. These findings highlight the need for an effective surveillance system, and for coordinated communication actions on cholera that target healthcare professionals and the populations living along the Oubangui River, to prevent and control cholera outbreaks in CAR.

Appendix 1 Additional tables for phylogenetic survey of seventh pandemic *Vibrio cholerae* O1 sublineages, Central African Republic.

Appendix 2 Additional information and methods used in phylogenetic survey of seventh pandemic *Vibrio cholerae* O1 sublineages, Central African Republic.
