# Supplementary material for: Seventh Pandemic Vibrio cholerae O1 Sublineages, Central African Republic
Source: Emerg Infect Dis. 2021 Jan;27(1):262–6. doi: 10.3201/eid2701.200375 (PMC7774542; doi:10.3201/eid2701.200375)
Supplement: Appendix 2 — Additional information and methods used in phylogenetic survey of seventh pandemic Vibrio cholerae O1 sublineages, Central African Republic. [file 20-0375-Techapp-s2.pdf]

Article DOI: https://doi.org/10.3201/eid3201.200375

Seventh Pandemic *Vibrio cholerae* O1 Sublineages, Central African Republic

Appendix 2

Antimicrobial Susceptibility Testing

Antimicrobial susceptibility was determined by the disk diffusion method, on Mueller-Hinton agar (Bio-Rad, https://www.bio-rad.com), in accordance with the guidelines of the Antibiogram Committee of the French Society for Microbiology (*1*). The following antimicrobial drugs (Bio-Rad) were tested: ampicillin, cefalotin, cefotaxime, streptomycin, chloramphenicol, azithromycin, sulfonamides, trimethoprim-sulfamethoxazole, vibriostatic agent O/129, tetracycline, nalidixic acid, ciprofloxacin, nitrofurantoin, polymyxin B, and colistin. *Escherichia coli* CIP 76.24 (ATCC no. 25922) was used as a control. The MICs of nalidixic acid and ciprofloxacin were determined by Etests (bioMérieux, https://www.biomerieux.com).

Whole-Genome Sequencing

We analyzed 30 *Vibrio cholerae* O1 biotype El Tor isolates from the Central African Republic (CAR) by whole-genome sequencing. Fourteen of these isolates were collected between 1997 and 2011 and were sequenced in a previous study (*2*). The 24 *V. cholerae* O1 biotype El Tor isolates sequenced here consisted of the remaining 16 isolates from CAR and included all isolates from 2016 and 8 isolates from neighboring countries, Chad and the Democratic Republic of the Congo (Appendix 1 Table 1).

Total DNA was extracted with the Maxwell 16-cell DNA purification kit (Promega, https://www.promega.com) in accordance with the manufacturer's recommendations. Whole-genome sequencing was carried out at the Biomics and PIBnet sequencing platforms of the Institut Pasteur, the genotyping and sequencing core facility of the Institut du Cerveau et de la Moëlle Epinière (Paris, France), or at Macrogen Korea (Seoul, South Korea), on Illumina

platforms (Illumina, https://www.illumina.com) generating 100–250 bp paired-end reads, yielding a mean coverage of 306-fold (minimum 61-fold, maximum 654-fold).

Additional Genomic Data

Raw sequence files from 1,164 seventh generation pandemic *V. cholerae* El Tor (7PET) genomes described by Weill et al. (*2*,*3*) and Irenge et al. (*4*) were downloaded from the European Nucleotide Archive (ENA; https://www.ebi.ac.uk/ena) and included in this study (Appendix 1 Table 1). Twelve assembled genomes also described by Weill et al. (*2*,*3*) were downloaded from GenBank (https://www.ncbi.nlm.nih.gov/genbank; Appendix 1 Table 1) and included in this study. We generated 100 bp overlapping simulated reads from the 12 assembled genomes with fasta_to_fastq.pl (https://github.com/ekg/fasta-to-fastq/blob/master/fasta_to_fastq.pl).

Genomic Sequence Analyses

The paired-end reads and simulated paired-end reads were mapped onto the reference genome of *V. cholerae* O1 El Tor N16961 (GenBank accession nos. LT907989 and LT907990) with Snippy v4.1.0/BWA-MEM v0.7.13 (https://github.com/tseemann/snippy). Single-nucleotide variants (SNVs) were called with Snippy v4.1.0/Freebayes v1.1.0 (https://github.com/tseemann/snippy) under the following constraints: mapping quality of 60, a minimum base quality of 13, a minimum read coverage of 4, and a 75% read concordance at a locus for a variant to be reported. An alignment of core genome SNVs was produced in Snippy v4.1.0 for phylogeny inference. Short reads were assembled with SPAdes version 3.1.0 (*5*).

In silico multilocus sequence typing (MLST) for *V. cholerae* was performed with MLST version 2.0. (https://cge.cbs.dtu.dk/services/MLST), on assembled sequences for the entire dataset (*6*). The various genetic markers were analyzed with BLAST version 2.2.26. against reference sequences of the O1 *rfb* gene, *ctxB*, *wbeT*, and the whole locus of VSP-II, as previously described (*2*,*3*,*7*).

The presence and type of acquired antimicrobial resistance genes (ARGs) or ARG-containing structures were determined with ResFinder version 3.1.0. (https://cge.cbs.dtu.dk/services/ResFinder), BLAST analysis against GI-15, Tn*7*, and SXT/R391 integrative and conjugative elements, and PlasmidFinder version 2.0.1. (https://cge.cbs.dtu.dk/services/PlasmidFinder). The presence of mutations in the genes

encoding resistance to quinolones (*gyrA*, *parC*), resistance to nitrofurans (*VC_0715* and *VC_A0637*), or restoring susceptibility to polymyxin B (*vprA)* was investigated by manual analysis of the sequences assembled de novo with BLAST (https://blast.ncbi.nlm.nih.gov), as previously described (*2*,*3*,*7*).

Phylogenetic Analysis

Repetitive (insertion sequences and the TLC-RS1-CTX region) and recombinogenic (VSP-II) regions in the alignment were masked (*2*). Putative recombinogenic regions were detected and masked with Gubbins version 2.3.4 (*8*). A maximum likelihood (ML) phylogenetic tree was built from an alignment of 9,964 chromosomal SNVs, with RAxML version 8.0.20., under the GTR model with 200 bootstraps (*9*). The final tree was rooted on the A6 genome and visualized with iTOL version 5 (https://itol.embl.de) or FigTree version 1.4.2. (http://tree.bio.ed.ac.uk/software/figtree).

Data Availability

Short-read sequence data were submitted to the ENA (http://www.ebi.ac.uk/ena), under study accession no. PRJEB36666 (see Appendix 1 Table 4 for accession nos.).

References

1. French Society for Microbiology, Antibiogram Committee. Press release 2006, January 2006 edition [cited 2020 Nov 30]. https://www.sfm-microbiologie.org/wp-content/uploads/2020/07/casfm_2006.pdf

2. Weill FX, Domman D, Njamkepo E, Tarr C, Rauzier J, Fawal N, et al. Genomic history of the seventh pandemic of cholera in Africa. Science. 2017;358:785–9. PubMed https://doi.org/10.1126/science.aad5901

3. Weill FX, Domman D, Njamkepo E, Almesbahi AA, Naji M, Nasher SS, et al. Genomic insights into the 2016-2017 cholera epidemic in Yemen. Nature. 2019;565:230–3. PubMed https://doi.org/10.1038/s41586-018-0818-3

4. Irenge LM, Ambroise J, Mitangala PN, Bearzatto B, Kabangwa RKS, Durant JF, et al. Genomic analysis of pathogenic isolates of *Vibrio cholerae* from eastern Democratic Republic of the Congo (2014–2017). PLoS Negl Trop Dis. 2020;14:e0007642. PubMed https://doi.org/10.1371/journal.pntd.0007642

5. Bankevich A, Nurk S, Antipov D, Gurevich AA, Dvorkin M, Kulikov AS, et al. SPAdes: a new genome assembly algorithm and its applications to single-cell sequencing. J Comput Biol. 2012;19:455–77. PubMed https://doi.org/10.1089/cmb.2012.0021

6. Larsen MV, Cosentino S, Rasmussen S, Friis C, Hasman H, Marvig RL, et al. Multilocus sequence typing of total-genome-sequenced bacteria. J Clin Microbiol. 2012;50:1355–61. PubMed https://doi.org/10.1128/JCM.06094-11

7. Oprea M, Njamkepo E, Cristea D, Zhukova A, Clark CG, Kravetz AN, et al. The seventh pandemic of cholera in Europe revisited by microbial genomics. Nat Commun. 2020;11:5347. https://doi.org/10.1038/s41467-020-19185-y

8. Croucher NJ, Page AJ, Connor TR, Delaney AJ, Keane JA, Bentley SD, et al. Rapid phylogenetic analysis of large samples of recombinant bacterial whole genome sequences using Gubbins. Nucleic Acids Res. 2015;43:e15. 10.1093/nar/gku1196 PubMed https://doi.org/10.1093/nar/gku1196

9. Stamatakis A. RAxML-VI-HPC: maximum likelihood-based phylogenetic analyses with thousands of taxa and mixed models. Bioinformatics. 2006;22:2688–90. PubMed https://doi.org/10.1093/bioinformatics/btl446

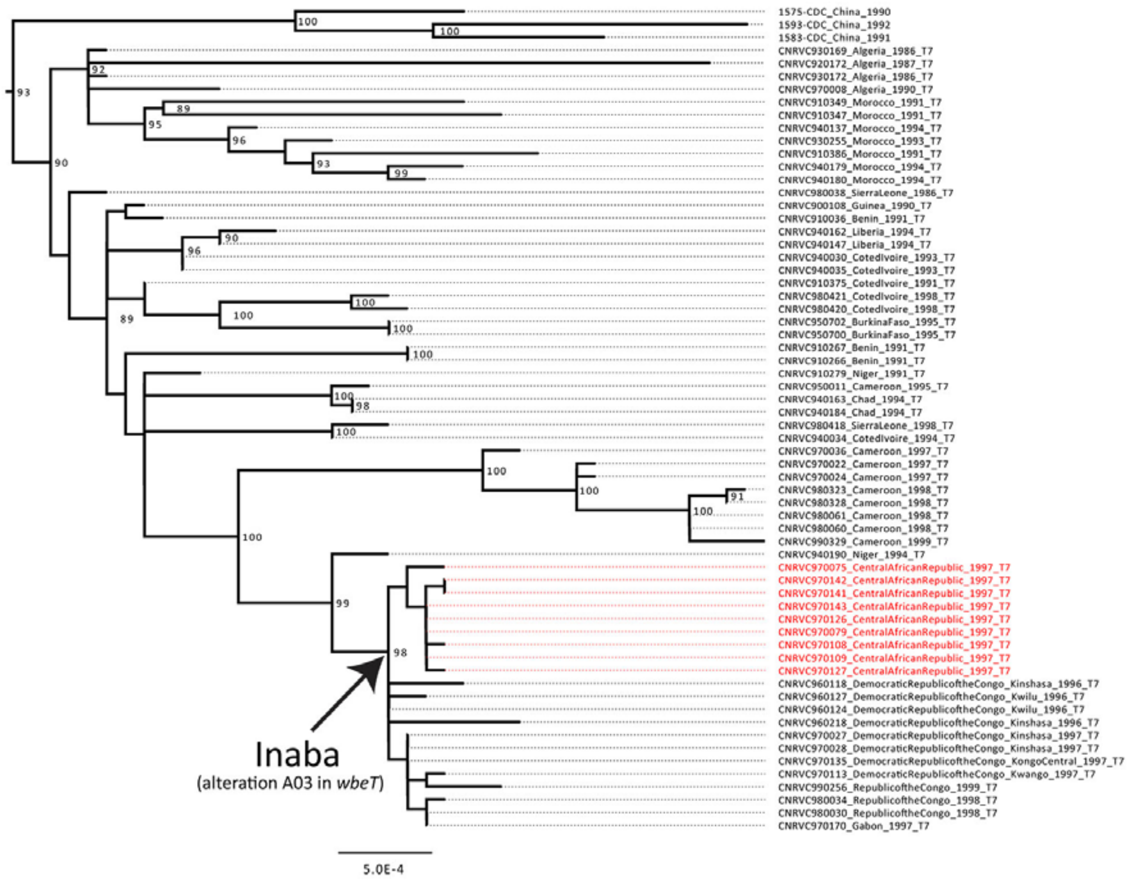

Appendix 2 Figure 1. Maximum likelihood phylogeny for the T7 seventh pandemic *Vibrio cholerae* El Tor sublineage. Bootstrap values ≥90% are shown at the branch nodes. The genomes from the Central African Republic are shown in red. Acquisition of the genetic variation of the *wbeT* (also named *rfbT*) gene implicated in the Inaba serotype, is indicated by an arrow.

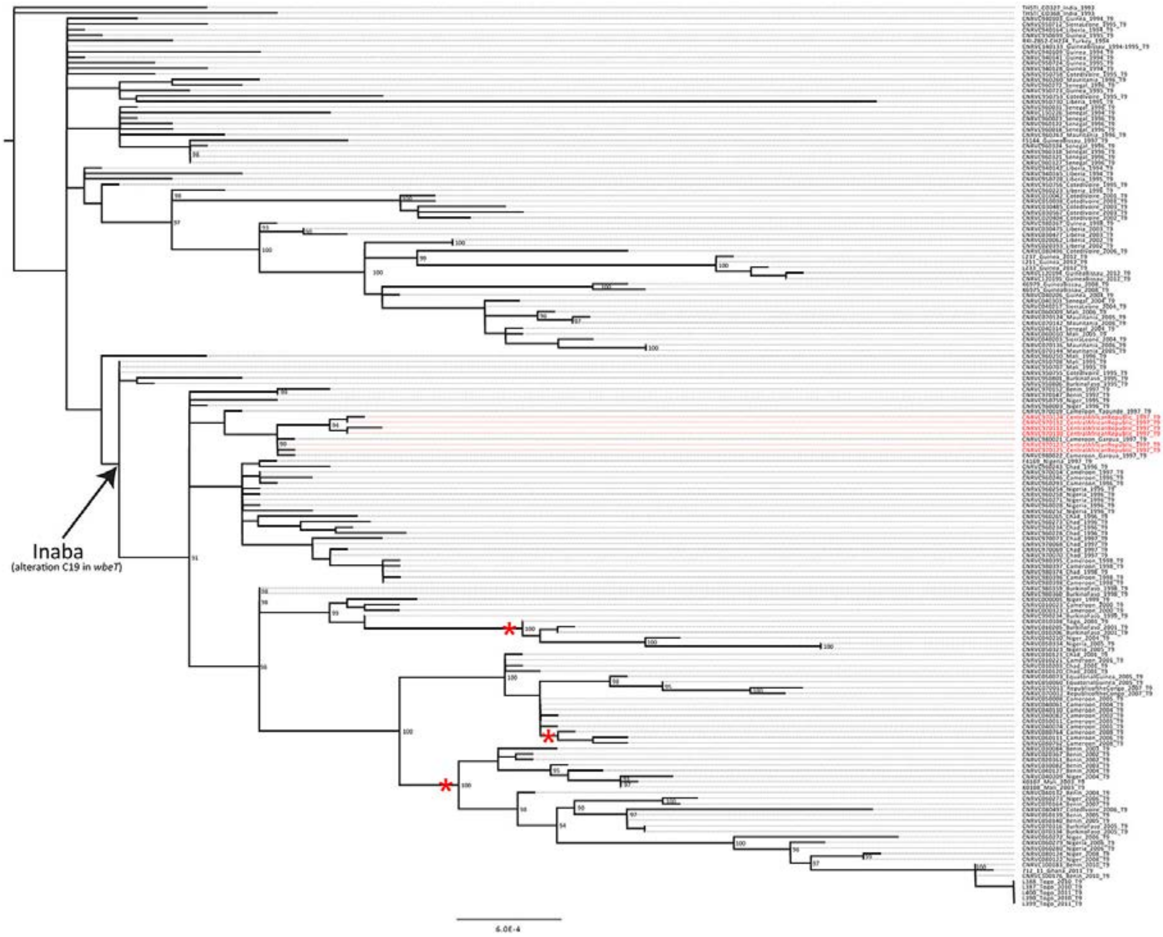

Appendix 2 Figure 2. Maximum likelihood phylogeny for the T9 seventh pandemic *Vibrio cholerae* El Tor sublineage. Bootstrap values >90% are shown at the branch nodes. The genomes from the Central African Republic are shown in red. Acquisition of the genetic variation of the *wbeT* (also named *rfbT*) gene implicated in the Inaba serotype, is indicated by an arrow. Red asterisks indicate the derived isolates are Ogawa revertants.

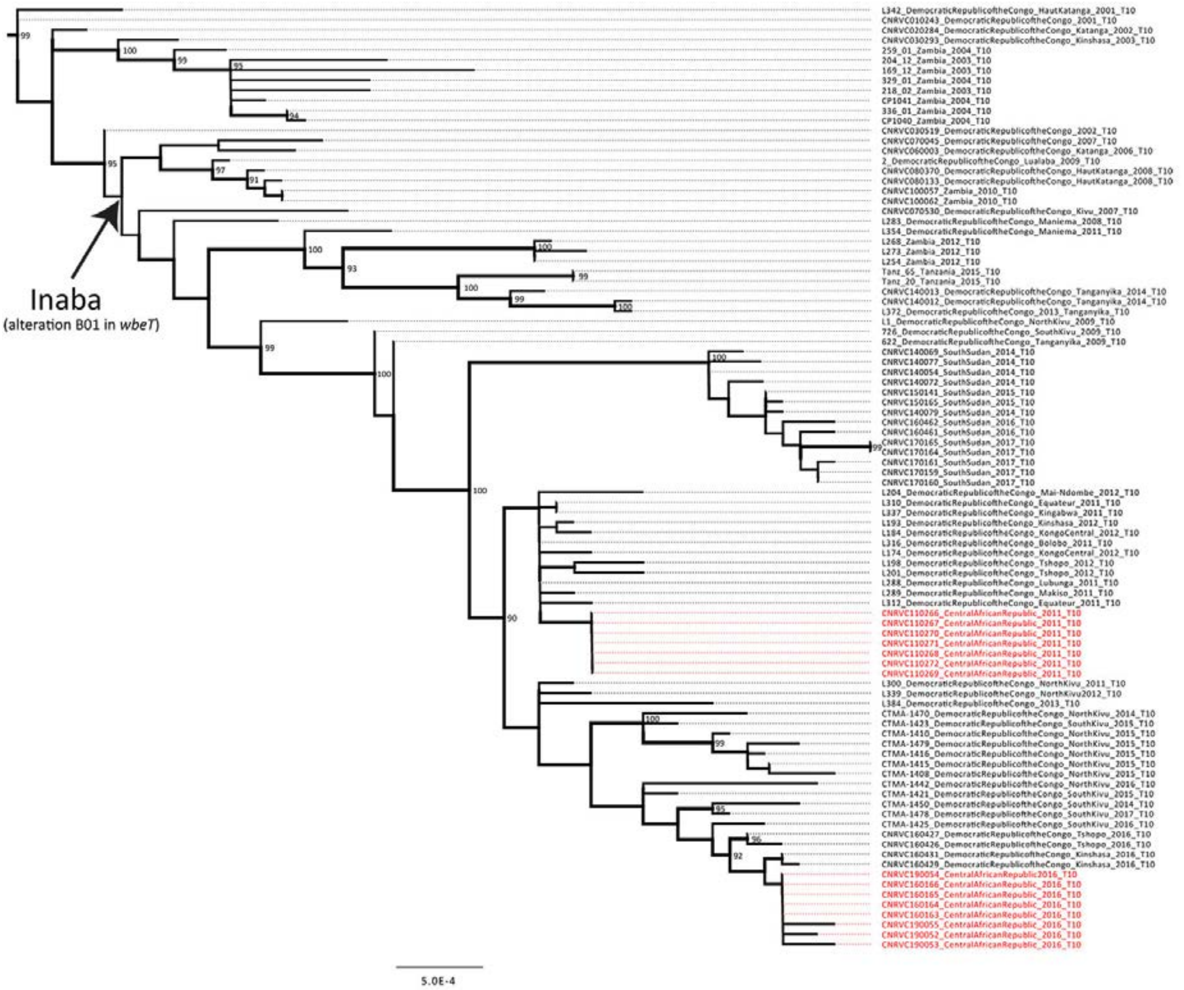

Appendix 2 Figure 3. Maximum likelihood phylogeny for the T10 seventh pandemic *Vibrio cholerae* El Tor sublineage containing sequence type 515 (ST515) *V. cholerae* O1 isolates from the Central African Republic. Bootstrap values greater than or equal to 90% are shown at the branch nodes. The genomes from the Central African Republic are shown in red. Acquisition of the genetic variation of the *wbeT* (also named *rfbT*) gene implicated in the Inaba serotype, is indicated by an arrow.
